# Targeted Disruption of the PME-1 Gene Causes Loss of Demethylated PP2A and Perinatal Lethality in Mice

**DOI:** 10.1371/journal.pone.0002486

**Published:** 2008-07-02

**Authors:** Silvia Ortega-Gutiérrez, Donmienne Leung, Scott Ficarro, Eric C. Peters, Benjamin F. Cravatt

**Affiliations:** 1 The Skaggs Institute for Chemical Biology and Department of Cell Biology, The Scripps Research Institute, La Jolla, California, United States of America; 2 The Skaggs Institute for Chemical Biology and Department of Chemistry, The Scripps Research Institute, La Jolla, California, United States of America; 3 The Genomics Institute for the Novartis Foundation, San Diego, California, United States of America; University of Cambridge, United Kingdom

## Abstract

**Background:**

Phosphoprotein phosphatase 2A (PP2A), a major serine-threonine protein phosphatase in eukaryotes, is an oligomeric protein comprised of structural (A) and catalytic (C) subunits to which a variable regulatory subunit (B) can associate. The C subunit contains a methyl ester post-translational modification on its C-terminal leucine residue, which is removed by a specific methylesterase (PME-1). Methylesterification is thought to control the binding of different B subunits to AC dimers, but little is known about its physiological significance in vivo.

**Methodology/Principal Findings:**

Here, we show that targeted disruption of the *PME-1* gene causes perinatal lethality in mice, a phenotype that correlates with a virtually complete loss of the demethylated form of PP2A in the nervous system and peripheral tissues. Interestingly, PP2A catalytic activity over a peptide substrate was dramatically reduced in *PME-1(−/−)* tissues, which also displayed alterations in phosphoproteome content.

**Conclusions:**

These findings suggest a role for the demethylated form of PP2A in maintenance of enzyme function and phosphorylation networks *in vivo*.

## Introduction

Reversible protein phosphorylation regulated by the coordinated action of protein kinases and phosphatases is an essential signaling mechanism for most cellular processes. Post-translational phosphorylation is predicted to occur on more than 30% of all eukaryotic proteins [1]. While phosphorylation is catalysed by more than 500 characterized kinases, a relatively small number of phosphatases are responsible for removing protein phosphorylation sites [2]. In mammalian cells, phosphoprotein phosphatase 2A (PP2A), together with PP1, account for >90% of the total serine/threonine phosphatase activity [3]. PP2A is a ubiquitously expressed multimeric protein that is highly conserved across eukaryotes ranging from yeast to human. PP2A is involved in numerous cellular processes, including signal transduction pathways that regulate mitogenic and survival signals, transcriptional and translational events, and the cell cycle [4], [5]. Moreover, several studies have identified alterations in specific PP2A subunits in human cancers, which has led to the hypothesis that PP2A serves as a tumor suppressor [6], [7].

Despite the central role that PP2A plays in cell signaling, the biochemical mechanisms that regulate PP2A activity in vivo are poorly understood. This is in part due to the complex composition and post-translational modification of PP2A. Structurally, it is a heterotrimeric complex typically consisting of a catalytic subunit (C), a scaffolding subunit (A or PR65) and one of an array of different regulatory subunits. In mammalian cells, the A and C subunits each have two isoforms (α and β), which share high sequence similarity. However, the most variable component of the holoenzyme is the regulatory subunit, which can belong to one of four different families termed B (or PR55), B′ (or B56 or PR61), B″ (or PR72) and B’’’ (or PR93/PR110). Each one of these gene families encodes multiple isoforms and splice variants. The different families of B subunits bind to overlapping regions on the AC dimer so their association to the core enzyme is mutually exclusive. Combinatorially, this variety of B subunits could generate as many as 70 different holoenzyme assemblies [4].

The current model for regulation of PP2A suggests that heterotrimers containing different regulatory subunits have distinct functions *in vivo* [8]. More specifically, B subunits, which exhibit cell- and tissue-restricted expression, are considered to be a major determining factor of substrate specificity by, for example, targeting PP2A to different intracellular locations [4], [9]. For example, the B subfamily has been shown to target PP2A to microtubules [10] while the B56 subfamily interacts with cyclin G and mediates PP2A functions in the Wnt/β-catenin and Erk signalling cascades [11]–[13]. Further support for this model stems from recent three-dimensional structural information obtained for the AC core enzyme [14] and the trimeric holoenzyme [15], [16]. The intrinsic flexibility of the A scaffolding subunit [14] as well as the AC core dimer, which experiences profound conformational rearrangements after binding of the regulatory subunit [15], [16], has been proposed to be important for binding different regulatory subunits and for performing catalysis on a wide variety of substrates.

A further level of PP2A regulation is provided by post-translational modification of the catalytic subunit, which can be phosphorylated on Tyr^307^, as well as on a threonine residue [17], [18]. In addition, a unusual type of reversible methylation occurs at the carboxyl group of the C terminal Leu residue (Leu^309^) [19]. Carboxylmethylation of Leu^309^ (referred to hereafter as “methylation”) is catalysed by a specific *S*-adenosylmethionine dependent methyltransferase, termed leucine carboxylmethyltransferase (LCMT) [20], [21]. The demethylation reaction is catalysed by a specific PP2A methylesterase (PME-1), which has been purified and molecularly characterized [22], [23]. Whereas phosphorylation of the catalytic subunit of PP2A inhibits enzyme activity [17], [18], a link between methylation of Leu^309^ and PP2A activity has remained contentious with different groups observing opposed effects [4], [19], [20], [24]. The current generally accepted view is that methylation does not affect phosphatase activity *in vitro*, suggesting instead that this modification indirectly regulates PP2A activity by modulating the binding of different regulatory B subunits to AC dimers [14], [25]–[30]. By affecting the association of the C subunit with regulatory subunits *in vivo*, methylation is predicted to alter the targeting of PP2A to certain substrates and, as a consequence, potentially impact a wide range of signalling pathways [26]. Consistent with this model, disruption of the major methyltransferase responsible for methylating PP2A in yeast leads to severe growth defects [26]. On the other hand, deletion of the yeast *PME-1* gene did not result in an observable cellular phenotype, even though PP2A methylation levels were enhanced [26]. More recent studies have proposed new models about the role of PME-1 in the regulation of PP2A. In the yeast, PME-1 seems to control the generation of active C subunit biogenesis and holoenzyme assembly [31]. In mammalian cells, and consistently with its predominant nuclear location, the major in vivo function of PME-1 has been proposed to be the stabilization of the inactive nuclear PP2A pool [32].

To more clearly understand the impact of methylation on PP2A function in mammalian biology, we have created mice that lack PME-1 by targeted gene disruption (PME-1(–/–) mice). Deletion of PME-1 resulted in post-natal lethality, a phenotype that correlated with a nearly complete loss of demethylated PP2A in most mammalian tissues. PP2A activity and brain phosphoproteome were also altered in PME-1(–/–) mice. These data indicate that dynamic methylation is required for proper PP2A function *in vivo*.

## Materials and Methods

### Generation of PME-1(−/−) Mice

The *PME-1* gene was obtained as part of a commercial BAC clone (Invitrogen). The gene disruption construct was generated using PCR-amplified 5′ and 3′ homologous recombination fragments surrounding exon 7, which codes for amino acids 134–185 (including the catalytic serine nucleophile Ser^156^) of the *PME-1* gene. The 5′ and 3′ homologous recombination fragments were subcloned into the *Not*I and *BamH*I sites (5′) and *Xho*I and internal *Hind*III sites (3′) of the pKO scrambler NTKV-1901 vector. Primers for 5′ homologous end: 5′-GC GCGGCCGC CAC TGG CAG ACA CTC TCT CGC-3′ (forward, with NotI site) and 5′-GC GGATCC CTC ACA GCT ATC TCC TTT ACC-3′ (reverse, with BamHI site). Primers for the 3′ homologous end: 5′-GC CTCGAG GAG ACT CAT ATT GGA AGC TGG-3′ (forward, with XhoI site) and 5′-CAA CAG GGC TGC TAA CAC AGG-3′ (reverse). Homologous recombinant 129SvJ embryonic stem cell clones were identified by Southern blot analysis, and two such clones were used to generate chimeric mice on a C57BL/6 background. Chimeras from one of the two clones gave germline transmission of the mutated gene. All mice used in this study were first or second generation offspring from intercrosses of 129SvJ-C57BL/6 *PME(+/−)*. All work performed in mice was done in accordance with the guidelines of the Institutional Animal Care and Use Committee of The Scripps Research Institute.

### Genotyping of PME-1(+/+), (+/−) and (−/−) mice

For Southern blotting, genomic DNA was digested with *Hind*III and separated on a 1% agarose gel. Fragments were transferred to nylon membrane and probed with 5′ external probe. An external ∼330 bp probe was generated by PCR using the *PME-1* genomic BAC clone as template and the following primers: 5′-CAG TTA GCT AGG ATG TGC-3′ (forward primer 3′), 5′-CCA GAG GAA GTA AAC AGG-3′ (reverse primer 3′), 5′-TCT GGT GGG CTT ATA CCG-3′ (forward primer 5′) and 5′-TTC TTT TCT GGT CTT GCT TCC-3′ (reverse primer 5′). ^32^P-labeled probe was hybridised overnight at 65°C.

PCR genotyping was performed using the following primers: *PME-1(+/+)* primer set: 5′-G GTG TCT TCC TCC AGC ACT C-3′ and 5′-CCA TAC CAG GGG ACC TCC TAC-3′; *PME-1(−/−)* primer set: 5′-GTC ACA GGG GCA AAA CTG TC-3′ and 5′-GCT CCC GAT TCG CAG CGC ATC-3′ that gave PCR products of 259 and 360 bp, respectively. After initial denaturation at 94°C for 4 min, PCR amplification was performed at a denaturing temperature of 94°C for 30 sec and followed by annealing at 60°C for 30 sec and extension at 72°C for 30 sec (35 cycles).

### Western Blotting

Tissue homogenates were centrifuged at 100,000*g* for 45 min at 4°C degrees to generate membrane and soluble fractions. Soluble fraction from each tissue was analysed by standard SDS-PAGE (10% NuPAGE Bis-Tris gel, Invitrogen) and western blotting procedures (incubation with the primary antibodies was carried out overnight at 4°C and with the secondary antibodies for 1 hour at room temperature). Polyclonal anti-PME-1 was generated in rabbit against a PME-1-GST fusion protein. Anti-PP2A structural (A), regulatory (B and B′) and catalytic (total, methylated and demethylated) subunit antibodies were from Upstate. An anti-phosphotyrosyl phosphatase activator protein (PTPA) antibody was obtained from Upstate and an anti phospho-PP2A (Tyr^307^) antibody was obtained from Santa Cruz. Immunoblots incubated with anti-tubulin (Sigma) were carried out as appropriate for loading control (not shown in the figures for the sake of clarity) and taking into account for band quantification as detailed in the corresponding legends.

### PP2A Activity Assays

Phosphopeptide substrate assay. Phosphatase activity of E18 tissue homogenates was determined using the phosphopeptide KRpTIRR as a substrate with a PP2A immunoprecipitation phosphatase assay kit (Upstate) following the manufactureŕs recommendations. Free PO_4_^2−^ generated was quantified by measurement of the absorbance at 595 nm after addition of malachite green-molibdate reagent. A standard curve with free phosphate was used to determine the amount of phosphate generated. *p* -Nitrophenylphosphate (*p*NPP) assay. Phosphatase activity was determined following similar protocols to those previously described [25], [33]. PP2A was immunoprecipitated with a PP2A antibody (Upstate) and protein A-agarose beads (Upstate) from 2 mg of E18 tissue homogenates. Washed beads were resuspended in assay buffer (50 mM Tris·HCl pH = 8.5, 1.0 mM DTT, 10 mM MgCl_2_ and 0.10 mg/mL BSA) and incubated in presence of 10 mM *p*NPP at 37°C for 20 min. The reaction was terminated by addition of an equal volume of Na_2_CO_3_ 1.0 M and absorbance was measured at 405 nm.

### Phosphoproteomic analysis of mouse brain tissue

E18 brains from either PME-1(+/+) or (−/−) mice were homogenized in Trizol (Invitrogen) and proteins isolated following the manufactureŕs directions. The protein pellet was reconstituted in 75 µL of 6M guanidine·HCl (Gu·HCl) with phosphatase inhibitors (Sigma) and solubilized at 60°C for 30 min. Debris was pelleted by centrifugation and protein concentration was measured in the supernatant using a Bradford-based protein assay (Biorad). One milligram of total protein was brought to pH 8 by addition of 100 mM NH_4_HCO_3_, reduced with 10 mM dithiothreitol by incubation for 45 min at 60°C and alkylated with 20 mM iodoacetamide for 45 min in the dark. Proteomes were diluted with 100 mM NH_4_HCO_3_ to a final Gu·HCl concentration of 1 M and digested with trypsin (Promega, Madison, WI) overnight at 37°C. As an internal standard, the peptide FLApTGDGAR was added to each extract. Peptides were desalted using SPE columns (Waters, Milford, MA), spiked with a second standard peptide (LIEDAEpYTAK), and concentrated by vacuum centrifugation to a volume of 50 µL. Reductive amination was performed as described [34], with modifications. Briefly, desalted peptides (50 µL) from PME-1(+/+) were added to 450 µL 1 M HEPES buffer (pH 7.5), mixed with 40 µL formaldehyde (4% in water), vortexed, and then mixed immediately with 40 µL of freshly prepared cyanoborohydride (260 mM). The mixture was vortexed again and then allowed to react for 10 min. Tryptic peptides from PME-1(−/−) were labelled in a similar manner using d2-formaldehyde and sodium cyanoborodeuteride (Sigma-Aldrich). Afterwards, peptides were desalted, combined and then subjected to strong cation exchange (SCX) chromatography [35]. SCX fractions containing phosphopeptides were then analyzed using an automated immobilized metal affinity chromatography (IMAC)-HPLC/MS platform [36] coupled to an LTQ Orbitrap mass spectrometer (San Jose, CA). The mass spectrometer was operated in data dependent mode such that the top 5 most abundant ions in each MS scan (resolution = 100,000) were subjected to MS/MS (collision energy = 35%). MS/MS were searched using the SEQUEST algorithm [37] against a sub-database of human, mouse and rat proteins derived from the NCBI non-redundant protein database.

## Results

### Targeted disruption of the PME-1 gene

**Figure 1:**
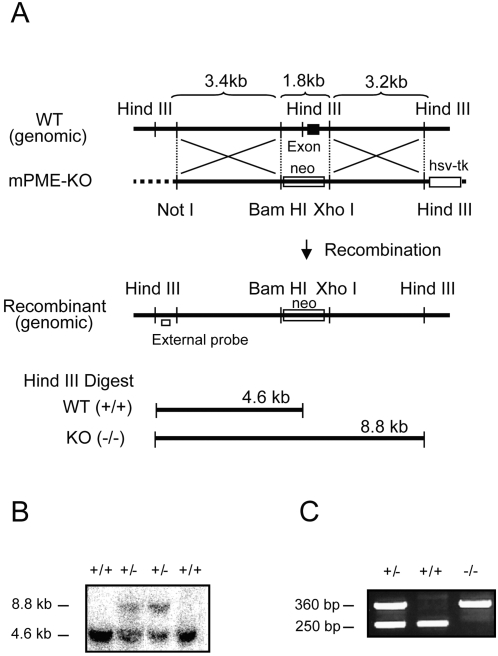
Generation of *PME-1(−/−)* mice. (A) Genomic structure surrounding the deleted exon-7 that encodes amino acids 134–185 of the PME-1 protein. Only relevant restriction sites are designated. (B) Southern blot analysis of *Hind*III-digested genomic DNA using the indicated external probe, where the 4.6 and 8.8 kb bands correspond to PME-1(+/+) and (−/−) genotypes, respectively. (C) PCR analysis of mouse genomic DNA, where the 250 and 360 bp bands correspond to PME-1(+/+) and (−/−) genotypes, respectively.

**Figure 2:**
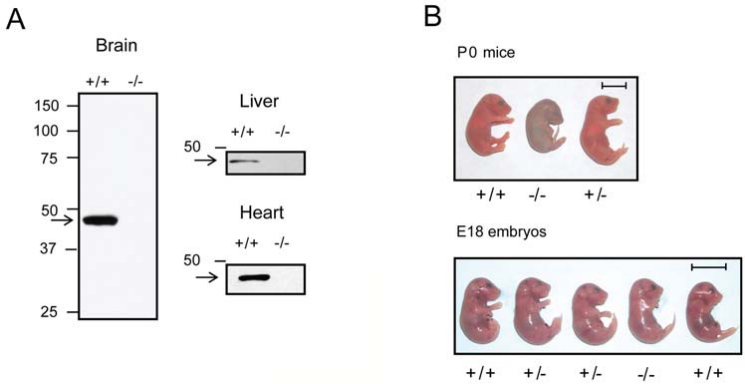
Deletion of PME-1 leads to postnatal lethality in mice. (A) Western blot analysis showing the absence of PME-1 protein in tissues from PME-1(−/−) mice. (B) upper panel, P0 littermates with genotypes shown [note dead pup for PME-1(−/−)]; lower panel, E18 embryos with genotypes shown. Scale bar, 1 cm.

To generate mice lacking PME-1 [PME-1(−/−) mice], the exon that encodes amino acids 134–185, including the conserved G_154_HSMG_158_ motif that contains the catalytic serine nucleophile, was deleted by homologous recombination (Fig. 1A). Two homologously recombined 129S6/SvEv embryonic stem cell clones were identified by Southern blotting (Fig. 1B) and used to generate chimeric mice on a C57BL/6 background. One of these clones gave germline transfer of the mutated gene (Fig. 1C) and was used to create PME-1(−/−) mice on an outbred background. Western blotting with an anti-PME-1 antibody confirmed the absence of PME-1 protein in PME-1(−/−) mice (Fig. 2A).

### Postnatal Lethality in PME-1(−/−) Mice

At the moment of birth, PME(−/−) mice showed no obvious abnormalities. However, these animals did not commence normal breathing or suckling behaviour and invariably died during first day of birth (Fig. 2B, upper panel). Further examination of animals on the day prior to birth (embryonic day 18.5) revealed the expected Mendelian frequency of genotypes [total pups: 128; PME(+/+) = 34 (26.6%), PME(+/−) = 64 (50%), PME(−/−) = 41 (24.2%)], with all genotypes appearing phenotypically normal. (Fig. 2B, lower panel). These data indicate that PME-1(−/−) mice develop normally in utero, but are incapable of surviving post-natal.

### PME-1 is the primary enzyme responsible for the in vivo demethylation of the catalytic subunit of PP2A

**Figure 3:**
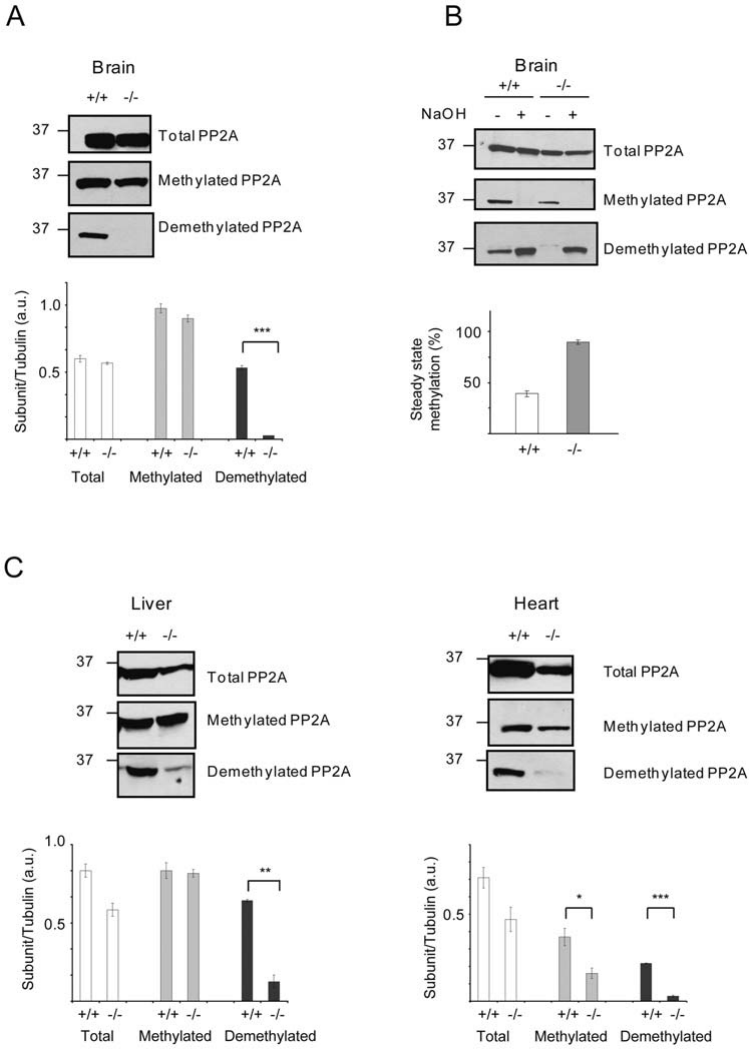
Impact of PME-1 deletion on the methylation state and expression level of the PP2A catalytic (C) subunit. (A) Expression of the different forms of the PP2A catalytic (C) subunit in brain tissue from PME-1(+/+) and (−/−) mice (harvested from E18 embryos). Western band quantification results are reported as their ratio over tubulin as loading control (not shown) as mean±standard error (SE) for at least four independent experiments. ***p<0.001, for PME(+/+) versus (−/−) samples. (B) Estimation of the percentage of steady-state methylation in brain tissue from PME-1(+/+) and -(−/−) E18 embryos. The fraction (x) of methylated C subunit in the original samples is defined as the ratio of the demethylated C subunit signal in untreated versus NaOH-treated samples. This fraction was used to calculate the steady-state methylation level (% = 100×[1−x]). The results are presented as mean values±SE of three independent experiments from which one representative gel is shown. (C) Expression of the different forms of the PP2A C subunit in liver and heart tissue from PME-1(+/+) and -(−/−) E18 embryos. Western band quantification results are means±SE for at least two independent experiments. *p<0.01, **p<0.005, ***p<0.001 for PME-1(+/+) versus (−/−) samples.

PME-1 has been described as the enzyme that specifically demethylates the catalytic subunit of mammalian PP2A based on *in vitro* data using either reconstituted systems [22] or in lysates from PME-1-expressing bacteria [23]. However, *in vivo* verification of this hypothesis has remained lacking. We compared the levels of methylated and demethylated PP2A in tissues from PME-1(+/+) and (−/−) mice using methylation-specific antibodies. Brain extracts from PME-1(−/−) mice contained essentially no demethylated form of PP2A (Fig. 3A). The loss of demethylated PP2A in brain tissue of PME-1(−/−) mice occurred without significant changes in total PP2A levels (Fig. 3A). The percentage of methylated and demethylated PP2A was estimated in brain tissue from PME-1(+/+) and (−/−) mice by comparing the signals of these proteins before and after treatment with strong base (0.1 M NaOH) to chemically cleave the methylester bond. These studies estimated that ∼40% and >95% of PP2A exists in the methylated state in PME-1(+/+) and (−/−) brains, respectively (Fig 3B). One surprising finding is the apparent lack of net increase of the methylated signal in PME-1(−/−) brains. One possible reason for this result could be the presence of another C terminal modification that hampers recognition by the antibody such as the phosphorylation at Tyr^307^ (although none of our attempts succeeded in providing direct evidence of its presence) or at Thr^304^, the importance of which has been recently suggested [38]. Alternatively, dissimilar systems may differ in their suitability for visualization of changes in methylation. In this regard, for example, no change in the methylation levels of C subunit was observed in the RNAi-mediated knocked down of PME-1 in HeLa cells [32].

Peripheral tissues from PME-1(–/–) mice, such as heart and liver, also showed a dramatic decrease in the levels of demethylated PP2A; however, some residual signals for this form of the enzyme could be detected in the absence of PME-1 (Fig. 3C). In peripheral tissues, a significant reduction in total PP2A levels was also observed (Fig. 3C).

### Expression levels of structural and regulatory subunits of PP2A in PME-1(+/+) and (−/−) mice

Ablation of the catalytic (C) subunit of PP2A causes disappearance of all structural and regulatory PP2A subunits in *Drosophila* [8]. Therefore, we studied whether the elimination of the demethylated pool of PP2A exerted any effect on the expression of either the scaffold (A) or regulatory (B and B′) subunits. In brain, no alterations in PP2A structural or regulatory subunits were observed in PME-1(−/−) mice (Fig. *S1*). The structural subunit PP2A A was modestly decreased in heart and liver of PME-1(−/−) mice, possibly reflecting the diminished levels of total PP2A C expression in these two tissues (Fig. 3C).

### Decreased PP2A activity in PME-1(−/−) tissues

**Figure 4:**
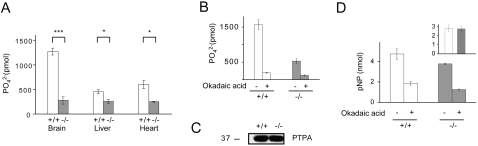
PP2A phosphatase activity in tissues from PME-1(+/+) and (−/−) mice. (A) PP2A was immunoprecipitated from different tissues of PME-1(+/+) and (−/−) E18 embryos and phosphatase activity towards the phosphopeptide substrate KRpTIRR was determined. Results are expressed in pmol of phosphate released per 10 min and 250 µg of protein and represent mean values±SE for at least three independent experiments (***p<0.001, *p<0.01 for PME-1(+/+) versus (−/−) samples). (B) Inhibition of phosphatase activity of brain immunoprecipitated PP2A by okadaic acid. Results represent mean values±SE of two independent experiments performed in duplicate (***p<0.001 for phosphatase activity in absence versus presence of okadaic acid). (C) PTPA expression in brains from PME-1(+/+) and (−/−) E18 embryos. Blot is representative of two independent samples. *D*, PP2A was immunoprecipitated from brain of PME-1(+/+) and (−/−) E18 embryos and phosphatase activity towards *p*-nitrophenylphosphate (*p*NPP) in presence or absence of okadaic acid was determined. Results are expressed in nmol of *p*-nitrophenol (*p*NP) released per 20 min and 2 mg of protein and represent mean values±SE for three independent experiments. Inset shows that the okadaic acid-sensitive fraction (obtained by subtraction of okadaic acid non-sensitive pNPPase activity from total pNPPase activity) of pNPPase activity (like the total pNPPase activity) was equivalent between PME(+/+) and (−/−) samples (*p<0.01 for phosphatase activity in absence versus presence of okadaic acid).

We next investigated the influence of the methylation state of PP2A on catalytic activity using a phosphopeptide substrate (KRpTIRR). Immunoprecipitated PP2A from PME-1(−/−) tissues showed significant reductions in phosphatase activity, which were most dramatic in brain tissue (∼4-fold reduction) (Fig. 4A). More than 80% of the phosphatase activity measured in this assay for both PME-1(+/+) and (−/−) tissues was inhibited by the PP2A inhibitor okadaic acid, confirming that it derived from the PP2A enzyme (Fig. 4B). Previous research has not revealed a direct link between the methylation state of PP2A and catalytic activity, although none of these studies were conducted with a fully methylated enzyme expressed in native mammalian tissues. Instead, it has been proposed that PP2A methylation might affect phosphorylation at Tyr^307^, which has been found to inactivate PP2A [17]. We therefore attempted to measure the contribution of phosphorylated PP2A to the total phosphatase activity in PME-1(+/+) and (−/−) brains by immunoprecipitation with an anti-phosphotyrosine antibody. Western blotting of the beads and the supernatant with an antibody that specifically recognizes phospho-tyrosine 307 of PP2A, as well as with antibodies for the different forms of the catalytic PP2A subunit (total, methylated and demethylated) did not yield any appreciable signal in immunoprecipitated samples from either PME-1(+/+) or (−/−) tissue. Instead, signals for total, methylated and demethylated PP2A were exclusively observed in the supernatant fraction (no signal was observed for phospho-tyrosine 307 PP2A in any sample). These data indicate that phosphorylation of Tyr^307^ is not a major steady-state modification on PP2A in brain tissue of PME-1(+/+) and (−/−) mice.

Another mode of regulation of PP2A occurs through binding to the PP2A phosphatase activator (PTPA) protein. PTPA is highly conserved from yeast to human and was initially named based on its ability to stimulate the basal level of phosphotyrosyl phosphatase activity of PP2A [39]–[41]. However, recent findings suggest that the more likely physiological function of PTPA is to reactivate the Ser/Thr phosphatase activity of an inactive form of PP2A [33], [42], [43]. Therefore, we analyzed whether the observed differences in PP2A activity between PME(+/+) and (−/−) tissues could be attributed to variations in the levels of PTPA. However, no significant changes in the levels of PTPA were observed in brain tissue from PME(+/+) and (−/−) mice (Fig. 4C).

Taken together, these data suggest that the decreased phosphopeptide phosphatase activity observed in PME-1(−/−) mice may be directly related to the absence of demethylated forms of the PP2A catalytic subunit. In order to establish whether the defective activity of PP2A was observed with other classes of substrates, we tested the activity of immunoprecipitated PP2A towards the small-molecule substrate *p*-nitrophenylphosphate (*p*NPP). Interestingly, no significant differences were observed in *p*NPPase activity in brain tissue from PME-1(+/+) and (−/−) mice (Fig. 4D). Similar results were obtained when the okadaic acid-sensitive portion of *p*NPPase activity was taken into account (Fig. 4D, inset), which accounted for approximately 60% of the total *p*NPPase activity in brain tissue from PME-1(+/+) and (−/−) mice These data suggest that the methylation state of PP2A selectively impacts the productive binding/recognition of phospho-peptide substrates rather than causing inherent defects in catalytic activity. A possibility that cannot be excluded from the present results is that the decrease in activity can be due to differential composition of the immunoprecipitates between the PME-1-(+/+) and (−/−) samples. Although statistically significant variations in the levels of the immunoprecipitated C subunit (total and methylated) were not detected by western blot (data not shown), their different composition cannot be ruled out, especially since this phenomenon has been widely described to influence PP2A activity [4], [8]–[13]. Therefore, instead of pursuing further characterization of in vitro activity towards a wide range of substrates (which may or may not reflect the physiological situation), we focused our efforts on profiling differentially phosphorylated proteins in the PME-1(+/+) and (−/−) mice.

### Analysis of candidate PP2A substrates by phosphoproteomic mapping in brain tissue from PME-1(+/+) and (−/−) mice

Methylation state of PP2A has been previously involved in the specific recognition of substrates [4], [26]. This fact, together with our data indicating impaired hydrolysis of a phosphopeptide substrate in tissues from PME-1(−/−) mice, suggested that this animal model might represent a suitable model to identify PP2A substrates for which PP2A demethylation (or methylation/demethylation cycling) is required. To begin to test this idea, we conducted a comparative phosphoproteomic mapping of brain tissue from PME-1(+/+) and (−/−) mice. Proteins were extracted from whole brain with Trizol, reduced, alkylated and digested with trypsin. To allow quantification, the mixture of peptides was isotopically labeled as detailed in the materials and methods section. After labeling, the PME-1(+/+) and (−/−) proteomes were combined, subjected to SCX chromatography [35] and analyzed using an automated IMAC-HPLC/MS platform [36] coupled to an LTQ Orbitrap mass spectrometer. Following this methodology, phosphopeptides present in both proteomes appear in the mass spectrum as doublets separated by 6 *m/z* units per modified primary amine group in the peptide. Phosphopeptides that are unique to one or the other proteome appear as singlets. After phosphopeptide identification using SEQUEST [37], the ratio between the area of the peaks of the heavy and light version of the same phosphopeptide was used for quantification. Reproducibility of the peptide extraction, derivatization and analytical methodology was ensured by analyzing three d_0_- and d_3_- labelled independent proteomes. The proteins identified as up- or down-regulated in at least two of three independent experiments with a difference between PME-1(+/+) and (−/−) samples of at least two fold were considered significant hits and were manually validated (Tables 1 and 2).

**Table 1:** Phosphoproteins increased in PME-1(−/−) brains

General class		Name, accession number (gi) and phosphorylated residue	Fold change^†^	Known to interact with PP2A
Cellular communication and signal transduction	Adaptor Molecules	Gab1 (10433473) (Ser 98)	2.5	Yes*^a^*
	GEF	Dock7 (Rac GEF) (12698087) (Ser 600)	2.5	No
	Protein kinases	Cdc2 (4502709) (Tyr 15)	2.7	Yes*^b^*
		SNIL (12643489) (Ser 437)	3.33	No
DNA replication		BM28 homolog (2183319) (Ser 27)	2.6	No
Transcription factors and transcriptional regulators		TF1B (3183181) (Ser 501)	3.3	No
		Tubby-superfamily protein (9502080) (Ser 1377)	2.8	No
Protein synthesis	Translation initiation complex	EIF5 (9910214) (Ser 10 and 227)	2.5	No
Structural/Cytoskeletal		Lamin B1 (6754556) (Ser 23)	3.0	No
Unkown		AK010820 (12846529) (Ser 235)	2.5	
		KIAA1757 (12698059) (Ser 39)	2.3	
		KIAA0386 (2224713) (Ser 21)	3.1	

**Table 2:** Phosphoproteins decreased in PME-1(−/−) brains

Cellular communication and signal transduction	Adaptor/Scaffold Molecules	X11 (423056) (Tyr 135)	0.3	No
		Neuronal protein 4.1 (2224617) (Ser 784)	0.4	No
		Palmitoylated membrane protein 2 (4885493) (Ser 42)	0.4	No
	Receptors	Mglu7 (547904) (Ser 900)	0.4	No
		Phosphacan (6755250) (Ser 276)	0.1	No
	GAPs	GAP120 (6753930) (Ser 225)	0.3	No
	Vesicle Trafficking	Syntaxin (9297065) (Ser 287 and 288)	0.3	Yes*^c^*
		Reticulon (12643485) (Ser 352)	0.4	No
Transcription factors and transcriptional regulators		RYBP (9790205) (Ser 201)	0.4	No
		PC4 AND SFRS1 (11024645) (Ser 106)	0.5	No
		Lbh (12052926) (Ser 163)	0.2	No
		MeCP2 (12083609) (Ser 80)	0.3	
		ZBP89 (12585539) (Ser 306)	03	No
Structural/Cytoskeletal		MAP1B (5174525) (Ser 1260)	0.4	Yes*^d^*
		Neurofilament protein (8393823) (Ser 766)	0.1	Yes*^e^*
	Actin binding proteins	Drebrin (2498314) (Ser 659)	0.3	No
		Drebrin-like (7304993) (Thr 296 and Ser 274)	0.3	No
		Synaptopodin (2654323) (Ser 19)	0.1	No
Metabolism		CTPU (12643330) (Ser 315 and 319)	0.5	No
Unkown		KIAA1582 (10047239) (Ser 515)	0.3	
		AK003611 (12834382) (Ser 57)	0.4	
		AK009886 (12844957) (Ser 43)	0.4	
		AK011522 (12847703) (Ser56)	0.3	
		ArfGAP protein (11691875) (Ser 360)	0.4	
Unclassified		GAP43 (8393415) (Ser 96)	0.3	Yes*^f^*
		HASPP28 (12018258) (Tyr 17)	0.3	

† Fold change is defined as the ratio between the area of the peaks in PME-1(−/−) to (+/+) samples.

See references *^a^* [54]; *^b^* [4], [47]; *^c^* [55]; *^d^* [44], [45]; *^e^* [46]; *^f^* [56].

Using the aforementioned criteria, we successfully sequenced 40 phosphopeptides corresponding to 38 proteins that differed between PME-1(+/+) and (−/−) samples. The majority of the identified proteins are known phosphoproteins (with the exception of phosphacan). Interestingly, in addition to detecting some known PP2A interacting proteins and/or substrates [e.g., MAP1B [44], [45], neurofilament protein [46] and Cdc2 [4], [47], we have also characterized previously unidentified phosphorylation sites as candidates for PP2A action. Identified phosphoproteins mainly fell into three categories based on predicted function: proteins involved in cellular communication, transcriptional regulation and cellular structure. A limited number of proteins with roles in the DNA replication and protein synthesis (BM28 homologue and the eukaryotic initiation factor 5, respectively) as well as metabolic functions (phosphorylcholine transferase B) were also identified. The phosphoserine/threonine proteins down-regulated in PME-1(−/−) (or phosphorylated on tyrosine residues, of which there were three examples) likely represent indirect consequences of alterations in PP2A activity in PME(−/−) mice. Conversely, those elevated in PME-1(−/−) brain tissue could be either substrates of a hyperactive kinase which activity is negatively regulated by PP2A or, alternatively, candidate direct substrates of PP2A.

A closer examination of the list of putative direct substrates of PP2A (i.e., phosphoproteins elevated PME(−/−) brain tissue) suggests some molecular and cellular hypotheses potentially related to the pre-mature death observed in PME-1(−/−) mice. Gab1 plays an essential role in several steps of mammalian development. For example, in Gab1(−/−) mice, migration of myogenic precursor cells is impaired and muscles in the diaphragm are missing [48]. Dock 7 plays a critical role in axon development [49]. B-type lamins are found in all cell types and are expressed throughout development. In the nucleus, lamin B1 binds directly to chromatin and histones and has a direct role in DNA synthesis. An essential role for lamin B was confirmed by the analysis of mice deficient in this protein, which die in the perinatal period with defects in lung and bone [50], [51]. Two additional proteins with altered phosphorylation in PME-1(−/−) mice, Cdc2 and the translation initiation factor 5 (eIF5), exert a broader influence over cellular processes by governing entrance into mitosis [47] and initiation of protein synthesis [52], [53], respectively.

## Discussion

The post-translational carboxylmethylation of the catalytic subunit of PP2A appears to exist in all eukaryotic organisms from yeast to human and, therefore, likely represents a key mechanism for regulating PP2A activity. Methylation has been hypothesized to influence the association of the PP2A heterodimer with different B regulatory subunits, which in turn control PP2A intracellular location and recognition of substrates. This model has been supported by various *in vitro* biochemical studies [14], [27], [28], [30] and genetic experiments in yeast [25], [26], [29]. The latter results illuminated an important role for the primary PP2A methyltransferase in survival, but yeast lacking the major PP2A methylesterase (*Ppe1*) were without apparent phenotypic defect [26]. The endogenous functions of methylated/demethylated forms of PP2A in mammalian systems have not yet been explicitly tested.

Here we have investigated the function of mammalian *PME-1* gene by deleting it from mice. *PME-1* gene deletion resulted in perinatal lethality, a phenotype that correlated with essentially complete loss of the demethylated form of PP2A in brain tissue. Further studies revealed that PME-1(−/−) brain tissue also possessed significantly reduced PP2A activity with phosphopeptide substrates and diminished quantities of PP2A holoenzyme complexes. To begin to assess the net biochemical and cellular effects of these changes in PP2A activity and complex assembly, we performed a comparative phosphoproteomic analysis of brain tissue from PME-1(+/+) and (−/−) mice. Several phosphoproteins were identified that exhibited either elevated or reduced signals in PME-1(−/−) brains, suggesting that the absence of demethylated PP2A invokes widespread alterations in phosphorylation networks. Collectively, we interpret these results to indicate that demethylated PP2A plays essential non-redundant functions that cannot be undertaken by the methylated pool of this protein. This statement implicitly assumes that methylated PP2A is the main, if not the unique, PME-1 substrate. However, the data available about the absolute substrate specificity of PME-1 are far for being conclusive. Therefore, the possibility of the existence of alternative substrates that could potentially play a role in the lethality and/or differential PP2A activity can not be unequivocally ruled out. Having said this, and even in the presence of other potential PME-1 substrates, it is clear that no other enzyme can undertake the role of PME-1 in PP2A demethylation, since non-methylated PP2A is virtually absent from PME-1(−/−) tissues. Considering the importance that has been attributed to the methylation state of PP2A, it seems logic to hypothesize that at least part of the effects are due to the disruption of the PP2A methylation state. Precisely how the absence of demethylated PP2A leads to reductions in enzyme activity or differential recognition of substrates remains unclear. Possible explanations could be that proper functioning and stability of PP2A complexes requires the dynamic ability to switch between methylated and demethylated forms during the substrate binding/catalytic cycle. In the absence of PME-1, this cycling would be blocked, resulting in an imbalanced accumulation of methylated forms of PP2A. Another (and not necessarily mutually exclusive) explanation is that PME-1 is, itself, a key component of PP2A complexes *in vivo*. Potentially consistent with this latter idea, PME-1 has been shown to regulate active PP2A C subunit generation and holoenzyme assembly [31] and to stably associate with “inactive” forms of PP2A complexes in tissues [32], [42], suggesting that this protein may confer a regulatory effect on PP2A activity through both binding interactions as well as catalysis. This final hypothesis could be directly tested by attempting to rescue the biochemical and cellular phenotypes observed in PME-1(−/−) mice with catalytically active and inactive (e.g., Ser^156^-to-Ala) forms of the PME-1 enzyme. Such next-generation studies should further refine our understanding of the evidently critical role that post-translational methylation plays in regulating PP2A activity *in vivo*.

## Supporting Information

Figure S1 Expression of PP2A structural and regulatory subunits (A, B, and B′) in PME-1(+/+) and (−/−) tissues. Tissues were harvested from E18 embryos.
(0.29 MB PDF)
