# Supplementary figures and images for: Targeted Disruption of the PME-1 Gene Causes Loss of Demethylated PP2A and Perinatal Lethality in Mice
Source: PLoS One. 2008 Jul 2;3(7):e2486. doi: 10.1371/journal.pone.0002486 (PMC2438471; doi:10.1371/journal.pone.0002486)

Figure S1

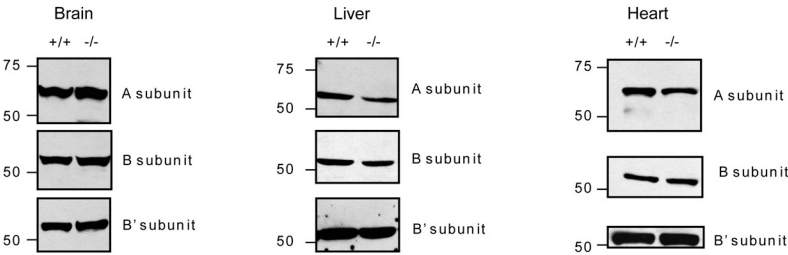

Supplement: Figure S1 — Expression of PP2A structural and regulatory subunits (A, B, and B′) in PME-1(+/+) and (−/−) tissues. Tissues were harvested from E18 embryos. (0.29 MB PDF) [file pone.0002486.s001.pdf]
